# Enhanced Nonlinear Optical Absorption in Fused-Ring Aromatic Donor–Acceptor–Donor Core Units of Y6 Derivatives

**DOI:** 10.3390/molecules30132748

**Published:** 2025-06-26

**Authors:** Xingyuan Wen, Tianyang Dong, Xingzhi Wu, Jiabei Xu, Xiaofeng Shi, Yinglin Song, Chunru Wang, Li Jiang

**Affiliations:** 1 School of Environment and Safety Engineering, North University of China, Taiyuan 030051, China; wenxingyuan191@gmail.com; 2 Beijing National Laboratory for Molecular Sciences, Key Laboratory of Molecular Nanostructure and Nanotechnology, Institute of Chemistry, Chinese Academy of Sciences, Beijing 100190, China; dongty@iccas.ac.cn (T.D.); crwang@iccas.ac.cn (C.W.); 3 University of Chinese Academy of Sciences, Beijing 100049, China; 4 School of Physical Science and Technology, Suzhou University of Science and Technology, Suzhou 215009, China; wuxingzhi@usts.edu.cn; 5 School of Physical Science and Technology, Soochow University, Suzhou 215123, China; 20224208057@stu.suda.edu.cn (J.X.); ylsong@hit.edu.cn (Y.S.)

**Keywords:** nonlinear optical absorption, Y6-derived fused-ring core units, Z-scan

## Abstract

This fundamental understanding of molecular structure–NLO property relationships provides critical design principles for next-generation optical limiting materials, quantum photonic devices, and ultrafast nonlinear optical switches, addressing the growing demand for high-performance organic optoelectronic materials in laser protection and photonic computing applications. In this study, it was observed that selenophene-incorporated fused D-A-D architectures exhibit a remarkable enhancement in two-photon absorption characteristics. By strategically modifying the heteroatomic composition of the Y6-derived fused-ring core, replacing thiophene (BDS) with selenophene (BDSe), the optimized system achieves unprecedented NLO performance. BDSe displays a nonlinear absorption coefficient (*β*) of 3.32 × 10^−10^ m/W and an effective two-photon absorption cross-section (*σ*_TPA_) of 2428.2 GM under 532 nm with ns pulse excitation. Comprehensive characterization combining Z-scan measurements, transient absorption spectroscopy, and DFT calculations reveals that the heavy atom effect of selenium induces enhanced spin–orbit coupling, optimized intramolecular charge transfer dynamics and stabilized excited states, collectively contributing to the superior reverse saturable absorption behavior. It is believed that this molecular engineering strategy establishes critical structure–property relationships for the rational design of organic NLO materials.

## 1. Introduction

With the continuous advancement of laser technology, nonlinear optics (NLO) has been applied in the fields of optical signal processing, telecommunication, sensing, photon generation and manipulation, and so on [1,2,3,4,5]. Compared with inorganic NLO materials, which have been deeply studied over the past few decades, organic materials are attracting an increasing amount of attention for their structural tunability, low preparation costs, and fast response times [6]. The degree of conjugation, charge transfer (CT), and assembled structure, which are usually mentioned in the donor or acceptor materials of organic photovoltaic devices (OPVs), have also been successfully proven to be the key factors to obtaining excellent NLO properties [7,8]. In particular, non-fullerene small molecular acceptors (SMAs) emerged as an excellent OPV in 2015: their unique Acceptor–π–Donor–π–Acceptor (A-π-D-π-A) structure attracted researchers to explore their NLO responses [9,10]. In our previous works, both fused-ring A-π-D-π-A-type electron acceptors and noncovalently fused-ring A-π-D-π-A-type electron acceptors have been reported to successfully achieve enhanced nonlinear optical absorption (NLA) [11]. However, a significant challenge is presented regarding thoroughly exploring the structure–property relationship of substantial acceptors to further enhance their nonlinear optical responses.

**Figure 1:**
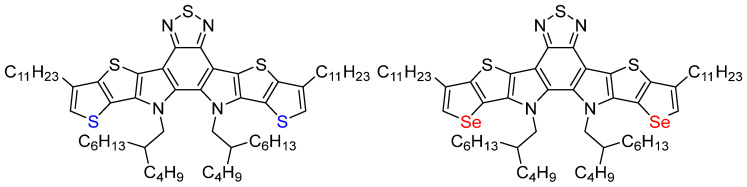
Molecular structures of BDS and BDSe.

Y6 and its derivatives with the A-DA’D-A structure have dominated as a star SMA in the field of OPVs since 2019 [12]. However, despite their remarkable optoelectronic properties demonstration in organic photovoltaic (OPV) devices, the nonlinear optical (NLO) characteristics of Y6 have not yet been systematically explored to date. Relevant research is urgently needed. In order to avoid strong absorption in the whole visible region, only the fused-ring DA’D core unit of Y6, 12,13-bis (2-butyloctyl)-3,9-diundecyl-12,13-dihydro-[1,2,5] thiadiazolo [3,4-e] thieno [2″,3″:4′,5′] thieno [2′,3′:4,5] pyrrolo [3,2-g] thieno [2′,3′:4,5] thieno [3,2-b] indole (BDS), was chosen to be the target instead of Y6 itself. In addition, two sulfur atoms in BDS are replaced with two selenium atoms to form BDSe, in which the two selenophenes are expected to enhance the nonlinear optical responses further (Figure 1). Firstly, the atomic radius of Se is larger than that of S, which causes the outer electrons of Se to be more spread out in space, resulting in a lower electron density [13]. Moreover, selenophene shows enhanced quinoidal character and weakened aromaticity because of the better orbital overlap in the π-conjugated systems and the more delocalized electron cloud [14]. Moreover, selenophene-fused SMAs exhibit significantly broadened absorption and a slightly down-shifted lowest unoccupied molecular orbital (LUMO) energy level. The stronger intermolecular interaction and higher electron mobility in selenophene-fused SMAs should also favor an enhancement in NLO properties [15].

In this context, a comparative study was performed to investigate the nonlinear optical behavior of BDS and BDSe in toluene solution using Z-scan technology. As expected, when using a 4 ns pulse width at 532 nm with different laser energies, BDSe exhibits superior nonlinear optical response compared to BDS. Both the nonlinear absorption coefficient (*β*) and two-photon absorption (TPA) cross-section (*σ*) of BDSe are nearly twice those of BDS. Moreover, the nonlinear absorption coefficient and TPA cross-sections of BDS and BDSe are inversely proportional to laser energies ranging from 1 to 38 μj. The maximum values of *β* occur when the laser energy drops to 1 μj, and are calculated to be 2.46 × 10^−10^ m/W for BDS and 3.32 × 10^−10^ m/W for BDSe. Meanwhile, the TPA cross-sections of BDS and BDSe were determined to be 1655.8 GM and 2428.2 GM, respectively. The nonlinear absorption coefficient and TPA cross-section are superior to those reported for organic small molecules. Transient absorption spectroscopy was performed to understand why the excited-state lifetime of BDSe was longer than that for BDS. Moreover, density functional theory (DFT) calculations revealed that BDSe possesses a higher dipole moment than BDS. All of the above factors resulted in a larger nonlinear optical response for BDSe than for BDS.

## 2. Results and Discussion

The powder samples of BDS and BDSe were purchased from Nanjing Zhiyan Technology Co., Ltd. (Nanjing, China), and characterized using ^1^H NMR (Figures S1 and S2) and high-resolution mass spectrometry (Figure S3). Solutions of BDS and BDSe in toluene with a concentration of 0.92 μmol/mL were obtained by dissolving 1.00 mg of BDS and 1.09 mg of BDSe in 1 mL toluene, respectively. The solutions were sonicated for 30 min before measurements.

**Figure 2:**
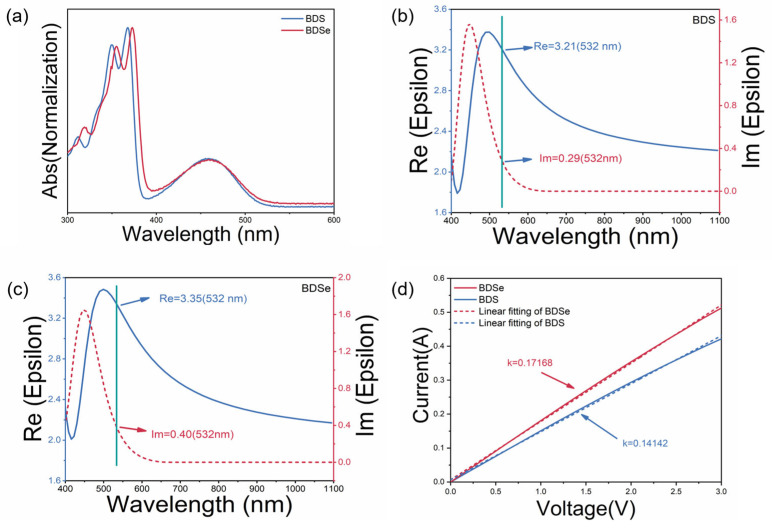
(**a**) UV–vis absorption spectra of BDS and BDSe in toluene solution; (**b**) dielectric constants of BDS; (**c**) dielectric constants of BDSe; (**d**) SCLC test results for BDS and BDSe.

First, UV-vis absorption spectra were obtained for BDS and BDSe in toluene solutions, as shown in Figure 2a. In the UV-vis absorption spectra, both BDS and BDSe molecules exhibit remarkably similar absorption profiles, with BDSe showing a slight redshift compared to BDS. Three distinct absorption peaks are clearly observable in the spectral curves of both compounds. The UV-vis spectral analysis reveals distinct electronic transitions in these compounds. The dominant absorption maxima centered at 370 nm correspond to n-π* electronic transitions, while the secondary peaks observed at 350 nm are characteristic of π-π* transitions. Notably, the broad absorption feature extending to approximately 460 nm attributed to intramolecular charge transfer (ICT) processes. This ICT behavior appears fundamentally linked to the molecular architecture, particularly the optimized acceptor–donor–acceptor (A-D-A) configuration inherent to both BDS and BDSe systems. The slight red-shifted absorption of BDSe relative to BDS arises from selenium’s heavy atom effect, strengthening intermolecular forces. These interactions are markedly evident in their thin-film spectra (Figure S4), where BDSe shows greater spectral broadening than BDS. At the wavelength of 532 nm, the absorption intensity of BDSe is higher than that of BDS, which is consistent with their Im values shown in Figure 2b,c.

The formula for calculating the molar absorption coefficient is

*ε*= *A*/*cb* (1)

where *A*, *c*, and *b* are the absorbance, sample concentration, and path length of the absorption cell, respectively. From this, it can be concluded that when the sample concentration and the path length of the absorption cell remain constant, the molar absorption coefficient is directly proportional to the absorbance. During testing, the concentration of the toluene solution for BDS and BDSe was 0.92 μmol/mL, and the optical path length of the absorption cell used was 1 cm. Therefore, absorbance can be used to represent the material’s absorption capacity. From the test results, it can be seen that the molar absorptivity of BDSe at 532 nm is 1.29 times that of BDS (Figure S5). The band gaps of BDS and BDSe were calculated using the Tauc plot equation:

(2) αhv12=Bhv−Eg

where *α* is the absorption coefficient, *h* is Planck’s constant, *ν* is the frequency, *B* is a constant, and *E*_g_ is the band gap [16,17]. The *Tauc* plots for BDS and BDSe, derived from the above formula, yielded band gaps corresponding to the absorption edges in Figure 2a, with values of 2.4910 eV and 2.4859 eV, respectively (Figure S6).

It has been verified that the polarization capabilities and electronic mobility of organic molecules are more favorable for enhancing NLO response. Both an ellipsometer and a space-charge-limited current (SCLC) were used on BDSe and BDS to determine their dielectric constants, which reflects the material’s polarization ability and electronic mobility [18,19]. The variation curves of dielectric constant as a function of wavelength (400–1100 nm) of BDSe and BDS are shown in Figure 2b,c, consisting of two components: The real part (*R*_e_) and the imaginary part (*I*_m_). The real part (*R*_e_) represents the relative dielectric constant of the material, describing its polarization ability. The curve of imaginary part (*I*_m_) closely resembles that of the UV-vis absorption spectrum. Considering the Z-scan measurements of nonlinear optical properties were conducted at 532 nm, the permittivity value at this wavelength is explicitly annotated in the figure. The *R*_e_ values (blue solid curves) for BDS and BDSe are 3.21 and 3.35, respectively, while the *I*_m_ values (red dotted lines) are 0.29 and 0.40, respectively. The results demonstrate that BDSe exhibits enhanced polarization response compared to BDS at the Z-scan measurement wavelength. In the *J*-*V* curves of BDS and BDSe, shown in Figure 2d, where the solid lines represent the linear fitting results, the slope (*k*) for BDS and BDSe is measured to be 0.14142 and 0.17168, respectively. The electronic mobility (μ) can be calculated using the following formula:

(3) $$\mu =\frac{k^{2}\times 8d^{3}}{9\times \varepsilon_{r}\times \varepsilon_{0}}$$

where *d*, $\varepsilon_{r}$, and $\varepsilon_{0}$ are the thickness, vacuum dielectric constant, and relative dielectric constant, respectively. As a result, the electronic mobility of BDSe is calculated to be 1.43 times that of BDS, indicating that BDSe has superior electronic mobility compared to BDS. Owing to its superior polarization ability and higher electronic mobility compared to BDS, BDSe is anticipated to exhibit a more remarkable third-order nonlinear optical response.

**Figure 3:**
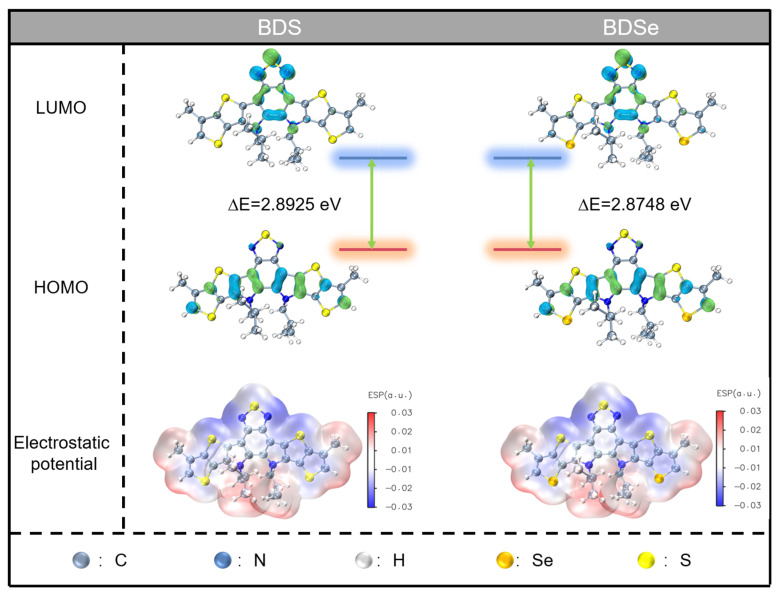
DFT calculation results of BDS and BDSe.

Density functional theory (DFT) calculations at the CAM-B3LYP/6-31G(d) level were conducted to investigate the frontier molecular orbital distributions of BDS and BDSe in toluene solution [20,21]. To simplify the computational model while maintaining chemical accuracy, the long alkyl chains in the molecular structures were replaced with methyl groups based on preliminary assessments, which showed that the side chains had negligible effects on the molecular conformation and electronic properties (see supporting Tables S2 and S3). As revealed in Figure 3, both compounds exhibit nearly identical molecular architectures and comparable frontier orbital distributions. The central 1,2,5-thiadiazole unit serves as an electron-accepting core, flanked by fused heterocyclic rings functioning as electron donors, collectively establishing a donor–acceptor–donor (D-A-D) structural motif. Theoretical analysis demonstrates similar electrostatic potential distributions between BDS and BDSe, although selenium substitution induces notable electronic effects. Replacing the sulfur atoms with selenium increases the calculated dipole moment from 6.0947 Debye (BDS) to 6.7975 Debye (BDSe). Concurrently, the theoretical band gap underwent a slight reduction from 2.8925 eV to 2.8748 eV following chalcogen substitution. This computational trend aligns with experimental observations from UV absorption spectroscopy, confirming the BDSe’s narrower band gap compared to BDS. The reduced band gap, combined with enhanced dipole moment, likely contributes to the improved nonlinear optical performance observed in BDSe derivatives, as narrower band gaps generally facilitate more efficient intramolecular charge transfer, a critical factor for nonlinear optical materials [22,23].

**Figure 4:**
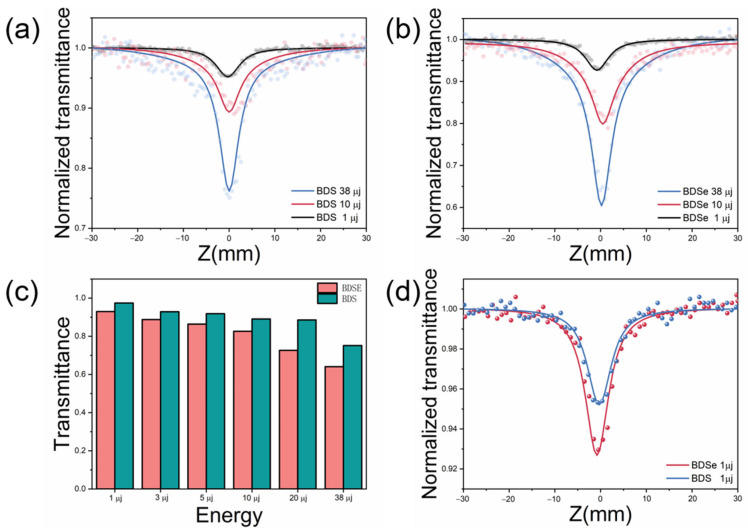
(**a**) Z-scan test results of BDS under different laser energies; (**b**) Zscan test results of BDSe under different laser energy; (**c**) comparison of Z-scan test results of BDS and BDSe under different laser energy; (**d**) Z-scan fitting results of BDS and BDSe under laser excitation with 1 μj of energy. The red, blue, and black circles in the figure represent the original test results at different energies, while the corresponding lines represent the fitting results.

The third-order nonlinear optical (NLO) properties of BDS and BDSe were investigated using the Z-scan technique with various laser energies at a wavelength of 532 nm and a 4 ns laser pulse duration. The obtained open-aperture Z-scan curves are shown in Figure 4 and Figure S7 with laser energy of 1, 3, 5, 10, 20, and 38 μj. In toluene solution, both BDS and BDSe displayed an obvious decrease in transmission independent of laser energy, which is characteristic of reverse saturable absorption. Notably, the nonlinear absorption increased with the decreasing of laser energy both for BDS (Figure 4a) and BDSe (Figure 4b). Moreover, under the same laser energy, the nonlinear absorption of BDSe is significantly stronger than that of BDS (Figure 4c). To enhance clarity, a plot (Figure S8) is added to illustrate the relationship between peak transmittance change and light intensity *I*_0_. The high linear transmittances (90% for BDS and 82% for BDSe) indicate that two-photon absorption may dominate the nonlinear absorption in both molecules. The decrease in nonlinear absorption with increased laser intensity shows that the nonlinear absorption of BDS and BDSe could be the sum effect of two-photon absorption and excited-state absorption with a certain degree of saturation on excited states. In this case, we further expand the third-order nonlinear absorption into two terms for easier estimation of the NLO capabilities in these molecules. All Z-scan data were fitted and analyzed using a modified saturation model based on Sheik Bahae’s theory, with dots representing the experimental data and solid lines representing the fitting results [24]. In this model, absorption of the material (*α*) is defined as

(4) $$\alpha =\alpha_{0}+\frac{\beta I}{\sqrt{1+{\left(\frac{I}{I_{S}}\right)}^{2}}}+\beta_{2}I$$

where *I* stands for incident laser intensity. Total absorption of the material consists of three terms, in which *α*_0_ stands for linear absorption while the original effective third-order nonlinear absorption is further divided into two parts (identified with the absorption coefficients *β* and *β*_2_). One experiences significant saturation characterized via saturation intensity *I*_s_; the other one (*β*_2_*I*) keeps the original form of intensity-dependent third-order NLO absorption. The laser intensity is modeled in situation of a typical focusing Gaussian beam in which its beam radius (*w*_z_) varies at different *z* positions as follows:

(5) $$w_{z}=w_{0}\sqrt{1+z^{2}/{z_{0}}^{2}}$$

where *w*_0_ denotes the minimum beam radius at focus and *z*_0_ represents the diffraction length of the beam. All the above calculation was programmed and the results were derived from numerical simulation on a PC.

The nonlinear absorption coefficients of BDS and BDSe obtained at different energy levels are listed in Table 1. Meanwhile, the effective two-photon absorption cross-section ($\sigma_{TPA}$) was calculated through *β* for estimation of nonlinear absorption capability of a single molecule, using the following formula:

(6) $$\sigma_{TPA}=h\omega \beta /N\;$$

From Table 1, it is evident that the nonlinear absorption coefficient (*β*) and TPA cross-section of BDSe are approximately 1.4 times those of BDS at any given laser energy. The larger dipole moment and enhanced intramolecular electron transfer capability provided by the Se atoms of BDSe are believed to be responsible for its superior NLO properties compared to BDS. Moreover, both the *β* values and *σ_TPA_* of BDS and BDSe increased with the decrease in laser energy. The highest *β* value observed for BDSe was 3.32 × 10^−10^ m/W, with a corresponding maximum *σ_TPA_* of 2428.2 GM, both achieved with 1 µj of laser energy. In contrast, the maximum *β* value for BDS was 2.46 × 10^−10^ m/W, and its largest *σ_TPA_* was 1655.8 GM (Figure 4d). In addition, Figure S9 lists the Z-scan data of BDS and BDSe obtained under 0.5, 0.3, and 0.15 µj of laser energy. The results show that the nonlinear absorption coefficient *β* does not change significantly.

**Figure 5:**
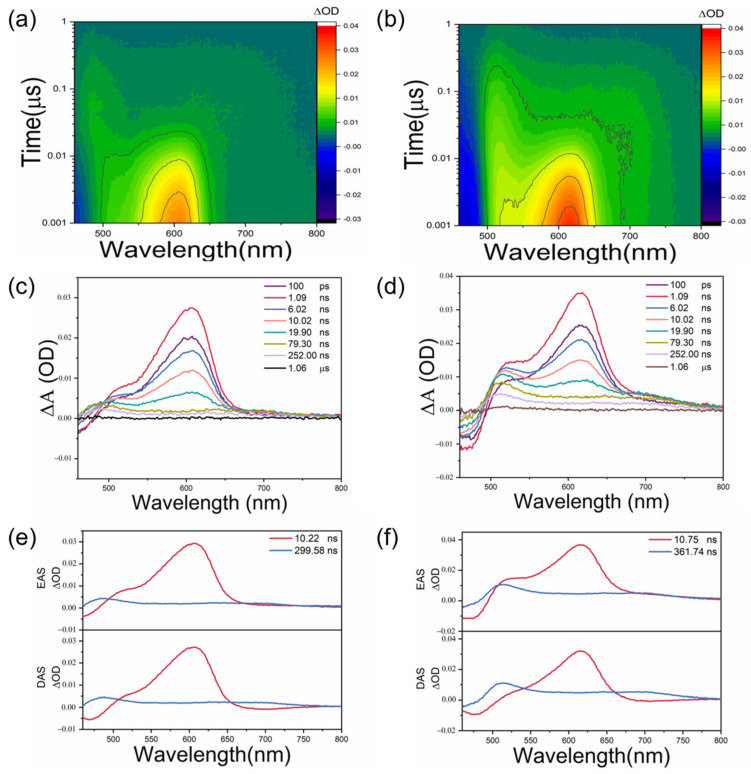
(**a**,**b**) Contour plot of ns-TA excited by BDS and BDSe at 430 nm; (**c**,**d**) selected TA spectra in contour plot corresponding to BDS and BDSe; (**e**,**f**) corresponding evolution-related spectra and decay-related spectra of BDS and BDSe.

To understand the dynamic process of photo-generated electron in BDS and BDSe, fluorescence decay curves were measured as shown in Figure S10. The fluorescence decay curves of BDS and BDSe both follow a biexponential decay curve. The lifetimes of the first component are 1.0 ns and 2.4 ns, while the lifetimes of the second component are 10.0 ns and 8.9 ns, respectively. The appearance of two components is due to the solvent relaxation of the small molecule in the solvent. Transient absorption spectra under excitation with a 430 nm laser were performed to obtain more accurate information, as shown in Figure 5a,b [25,26]. From the two similar transient absorption contour plots, it is evident that both BDS and BDSe possess a triplet state [27]. The spectral evolution exhibits an initial negative signal, which is subsequently followed by positive signals. The emergence of these positive signals stems from the interplay between excited-state absorption and ground-state bleaching (GSB). Notably, the negative signal persists over time rather than disappearing, indicating the presence of a long-lived GSB. This observation suggests that both materials undergo intersystem crossing to a triplet state upon excitation. Furthermore, the excited-state absorption for both BDS and BDSe evolves with time, with a strong absorption observed at 520 nm and 600 nm within 10 ns for both materials. However, beyond 10 ns, the absorption at 600 nm gradually diminishes and eventually disappears. Kinetic analyses affirm that both molecules possess a long-lived excited state, which further confirms the presence of triplet states in BDS and BDSe. Additionally, a slight redshift can be observed within a short timescale in the transient absorption spectra; this is attributed to the solvation relaxation of small molecules in the solvent. This observation is consistent with the results from the fluorescence decay curves (Figure S11). For a more precise analysis, global fitting of the obtained spectra was conducted using Glotaran 1.5.1 software, as shown in Figure 5e,f [28]. The fitting data unveiled two primary components: BDS exhibited lifetimes of 10.22 ns and 299.58 ns, whereas BDSe displayed lifetimes of 10.75 ns and 361.74 ns. The corresponding evolution-associated spectra (EASs) and decay-associated spectra (DASs) provide additional support for these findings. Importantly, the triplet-state lifetime of BDSe is longer than that of BDS, which is advantageous for enhancing nonlinear optical properties. This is corroborated by the superior performance of BDSe in the Z-scan results. A photophysical model was also established to illustrate these observations, as shown in Figure S12.

For a comparative analysis, Table 2 lists the nonlinear absorption coefficients and two-photon absorption cross-sections of BDS and BDSe alongside those of several other reported organic molecules measured under similar conditions. The exceptional NLO performance of BDS and BDSe is evident from the data presented.

## 3. Materials and Methods

### 3.1. Materials

The powder samples of BDS and BDSe were purchased from Nanjing Zhiyan Technology Co., Ltd. The toluene solvent was purchased from Sigma Aldrich (St. Louis, MO, USA).

### 3.2. Instrumentation

#### 3.2.1. UV-Vis

Use UH4150 spectrophotometer (Hitachi, Tokyo, Japan) to record the UV–visible absorption spectrum of the solution (toluene) and obtain the spectral absorption characteristics of the sample molecules.

#### 3.2.2. The Fluorescence Decay Curve

The fluorescence decay curve was obtained through FLS980 testing to study the dynamic processes of sample excitation and emission, and obtain the fluorescence lifetime of the sample.

#### 3.2.3. Transient Absorption

Ultrafast Transient Absorption Spectroscopy measurements (Ultrafast System, Helios and EOS) were measured at the Institute of Physics and Chemistry, Chinese Academy of Sciences. Similar description can be found elsewhere. The test samples are BDSs and BDS toluene solution, with a concentration of 0.92 μ mol/mL, placed in a colorimetric dish with a 1mm optical path. Briefly, the femtosecond laser (Coherent Inc., Santa Clara, CA, USA) delivered 25 fs pulses at 1 kHz and the output was split for white-light continuum generation. The excitation wavelength was obtained at a tunable optical parametric amplifiers (TOPAS-C, Light Conversion, Coherent Inc., Santa Clara, CA, USA). The specific excitation wavelength and power have been described in the text. The continuum was used as a broadband optical probe from the near-UV to the near-IR. Probe from 350 nm to 750 nm was generated by focusing the fundamental laser beam onto a 3 mm CaF2 plate (Coherent Inc., Santa Clara, CA, USA), which was oriented and continuously shifted in perpendicular directions. The near-IR probe was generated by focusing on a YAG crystal. The TA spectrum was calculated from consecutive pump-on and pump-off measurements and averaged over 400 shots. The nanosecond transient absorption is measured by the EOS detection system, wherein the super-continuous nanosecond laser is used, and the photonic crystal is excited by the Nd:YAG laser to produce 1 kHz broadband detection light, the detection spectrum covers 360–1750/2250 nm, and the pulse width is less than 1 ns. All samples used for TA measurements had an absorbance of about 0.7 OD (in quartz cuvette with 1 mm optical path) at their maximum wavelength of steady-state absorption, and solution sample concentrations were about 10^−4^ M. Steady-state absorption spectra of the samples were employed before and after every measurement to ensure that no remarkable photodegradation occurred during TA measurement.

#### 3.2.4. Z-Scan Technology

In the Z-scan technology, the light source origin in an optical parametric amplifier (OPA, ORPHEUS, Light Conversion, GRACE laser, Beijing, China) and Q-switched Nd: YAG laser (TINY-100, GRACE laser, Beijing, China) were used to obtain 4 ns (FWHM) pulses of 532 nm.

## 4. Conclusions

In this study, the third-order nonlinear optical properties of a fused-ring DAD core unit derived from Y6 were investigated using Z-scan techniques at 532 nm. With the replacement of two thiophenes of the fused-ring DAD core (BDS) with two selenophenes to form BDSe, the two molecules exhibited remarkable reverse saturable absorption characteristics under 4 ns pulse laser excitation. Notably, BDSe demonstrated a significantly superior nonlinear response compared to BDS due to the enhanced quinoidal character and improved orbital overlap in π-conjugated systems. Specifically, at a laser energy of 1 μj, the nonlinear absorption coefficient (*β*) of BDSe in toluene solution was 3.32 × 10^−10^ m/W, and the corresponding two-photon absorption cross-section (*σ*_TPA_) was calculated to be 2428.2 GM. These values were twice those of BDS in toluene solution under the same conditions, at 2.46 × 10^−10^ m/W and 1655.8 GM, respectively. Dielectric constant measurements, space-charge-limited current (SCLC) analyses, and DFT calculations supported that the larger dipole moment resulting from the Se atoms in BDSe led to enhanced nonlinear absorption compared to BDS. Transient absorption spectroscopy further confirmed that BDSe possessed a longer triplet-state lifetime than BDS, which is also advantageous for enhancing nonlinear optical properties. In conclusion, this study offers valuable insights into the design and optimization of organic nonlinear optical materials for future applications.

## Figures and Tables

**Table 1:** The values of *β*, $\sigma_{TPA}$, *γ*, *β*_2_, and *Is* of BDS and BDSe with different laser energies.

Laser Energy		1 μj	3 μj	5 μj	10 μj	20 μj	38 μj
BDS	*β* (m/W)	2.46 × 10^−10^		1.46 × 10^−10^			
	*σ*_TPA_ (GM)	1655.8		982.7			
	*γ* (esu)	2.67 × 10^−30^		1.58 × 10^−30^			
	*β_2_* (m/W)	2.75 × 10^−11^		3.15 × 10^−11^			
	*Is* (W/m^2^)	12.7 × 10^10^		9.74 × 10^10^			
BDSe	*β* (m/W)	3.32 × 10^−10^		2.21 × 10^−10^			
	*σ*_TPA_ (GM)	2428.2		1616.4			
	*γ* (esu)	3.92 × 10^−30^		2.61 × 10^−30^			
	*β_2_* (m/W)	8.14 × 10^−11^		7.26 × 10^−11^			
	*Is* (W/m^2^)	18.8 × 10^10^		18.8 × 10^10^			

**Table 2:** Comparison of NLO absorption coefficient in this work with that in the published literature.

Sample	Solvent	*Β* × 10^−10^ (m/W)	Reference
C_60_	Tol	0.01863	[29]
TrSR1	IPA	2	[30]
TrSR2	IPA	6.3	[30]
A	CH_2_Cl_2_	−0.443	[31]
B	CH_2_Cl_2_	1.76	[31]
P1-PT	1,1,2,2-tetrachloroethane	4.5	[32]
P2-PT	1,1,2,2-tetrachloroethane	7.9	[32]
BTH-1	DMSO	0.0016	[33]
BTH-2	DMSO	0.012	[33]
C_25_H_15_NS	DMF	0.0011	[34]
Nan-1	DMSO	0.00054	[35]
Py-Fc	CH_2_Cl_2_	0.0079	[36]
Py-PF	Tol	0.0345	[37]
HelFe	ACN	0.0352	[38]
NOC6F-1	Tol	0.057	[11]
NOC6F-2	Tol	0.010	[11]
BDS	Tol	2.46	This Work
BDSe	Tol	3.32	This Work

## Data Availability

The raw data supporting the conclusions of this article will be made available by the authors on request.

## Supplementary Materials

The following supporting information can be downloaded at https://www.mdpi.com/article/10.3390/molecules30132748/s1: Figure S1: The ^1^H NMR of BDS; Figure S2: The ^1^H NMR of BDSe; Figure S3: High-resolution mass spectrometry of BDS and BDSe; Figure S4: UV–visible absorption spectroscopy of BDS and BDSe films; Figure S5: Ratio of molar absorptivity of BDSe and BDS; Figure S6: Tauc plots of BDS and BDSe; Figure S7: Z-scan fitting results of BDS and BDSe under laser excitation with ns pulse width; Figure S8: The relationship between peak transmittance change and light intensity (*I*_0_) in Z-scan test results of BDS and BDSe; Figure S9: Z-scan test results of BDS and BDSe under excitation of ns pulse width laser with different energy excitations; Figure S10: The fluorescence decay curves of BDS and BDSe; Figure S11: Transient absorption spectra of BDS and BDSe; Figure S12: Schematic of typical photophysical processes; Table S1: Electronic transition types of BDS/BDSe molecules and their corresponding excitation energy calculations; Table S2: Cartesian coordinates of BDS; Table S3: Cartesian coordinates of BDSe.
