# Supplementary material for: Enhanced Nonlinear Optical Absorption in Fused-Ring Aromatic Donor–Acceptor–Donor Core Units of Y6 Derivatives
Source: Molecules. 2025 Jun 26;30(13):2748. doi: 10.3390/molecules30132748 (PMC12251174; doi:10.3390/molecules30132748)
Supplement: Supplementary file 1 [file molecules-30-02748-s001.zip › molecules-3610215-supplementary.pdf]

Supporting Information

Enhanced Nonlinear Optical Absorption in Fused-Ring Aromatic Donor–Acceptor–Donor Core Units of Y6 Derivatives

Xingyuan Wen [1,2,†], Tianyang Dong [2,3,†], Xingzhi Wu [4], Jiabei Xu [5], Xiaofeng Shi [1,*], Yinglin Song [5], Chunru Wang [2,3] and Li Jiang [2,3,*]

[1] School of Environment and Safety Engineering, North University of China, Taiyuan 030051, China; wenxingyuan191@gmail.com
[2] Beijing National Laboratory for Molecular Sciences, Key Laboratory of Molecular Nanostructure and Nanotechnology, Institute of Chemistry, Chinese Academy of Sciences, Beijing 100190, China; dongty@iccas.ac.cn (T.D.); crwang@iccas.ac.cn (C.W.)
[3] University of Chinese Academy of Sciences, Beijing 100049, China
[4] School of Physical Science and Technology, Suzhou University of Science and Technology, Suzhou 215009, China; wuxingzhi@usts.edu.cn
[5] School of Physical Science and Technology, Soochow University, Suzhou 215123, China; 20224208057@stu.suda.edu.cn (J.X.); ylsong@hit.edu.cn (Y.S.)
* Correspondence: xiaofeng_shi1987@163.com (X.S.); jiangli@iccas.ac.cn (L.J.); Tel.: +86-351-3920934 (X.S.); +86-10-82624962 (L.J.)
† These authors contributed equally to this work.

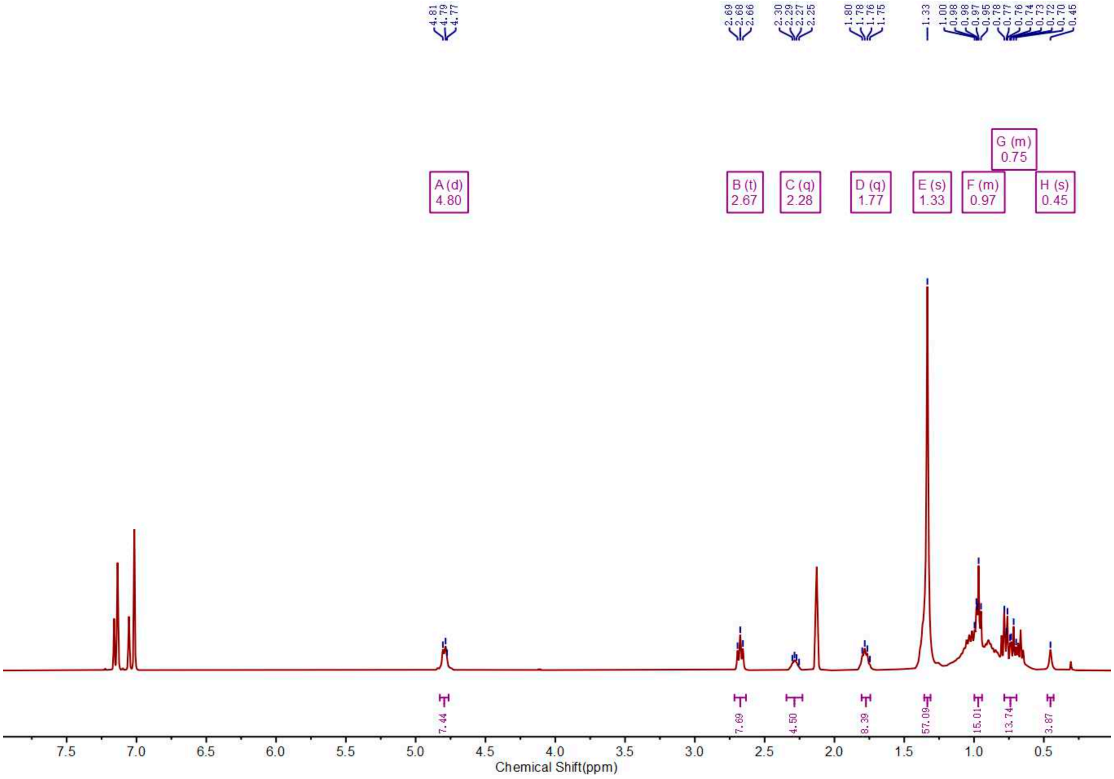

Figure S1. The ^{1}H NMR of BDS.

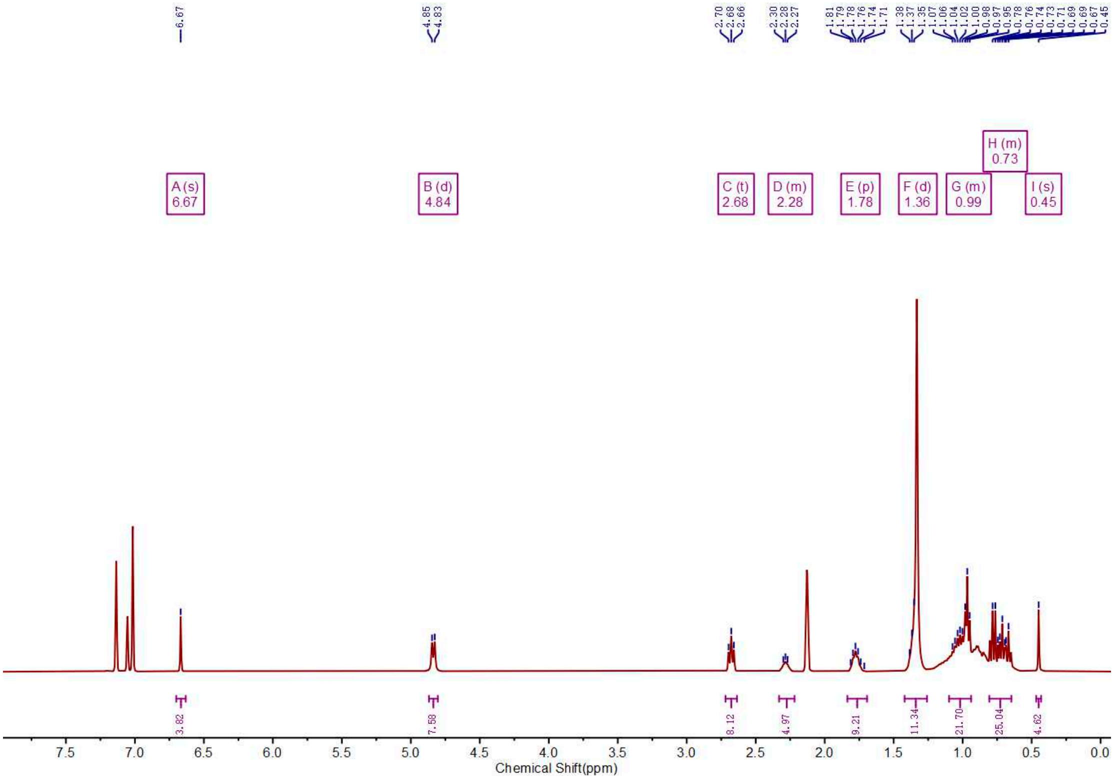

Figure S2. The ^{1}H NMR of BDSe.

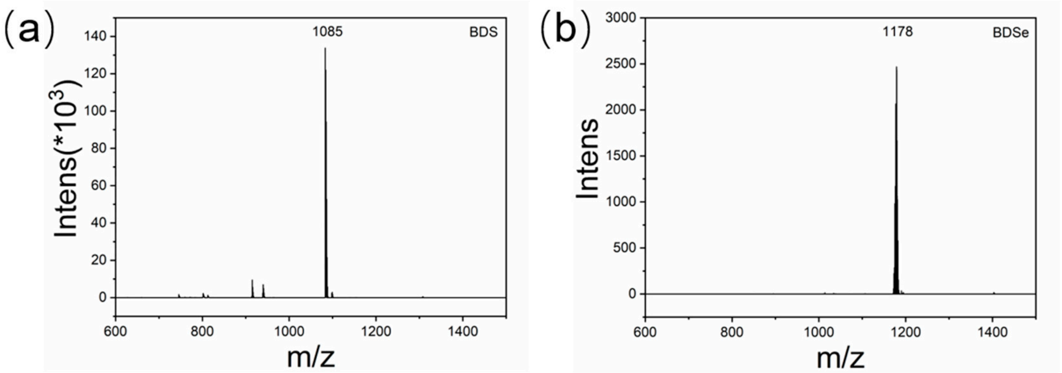

Figure S3. High resolution mass spectrometry of BDS and BDSe.

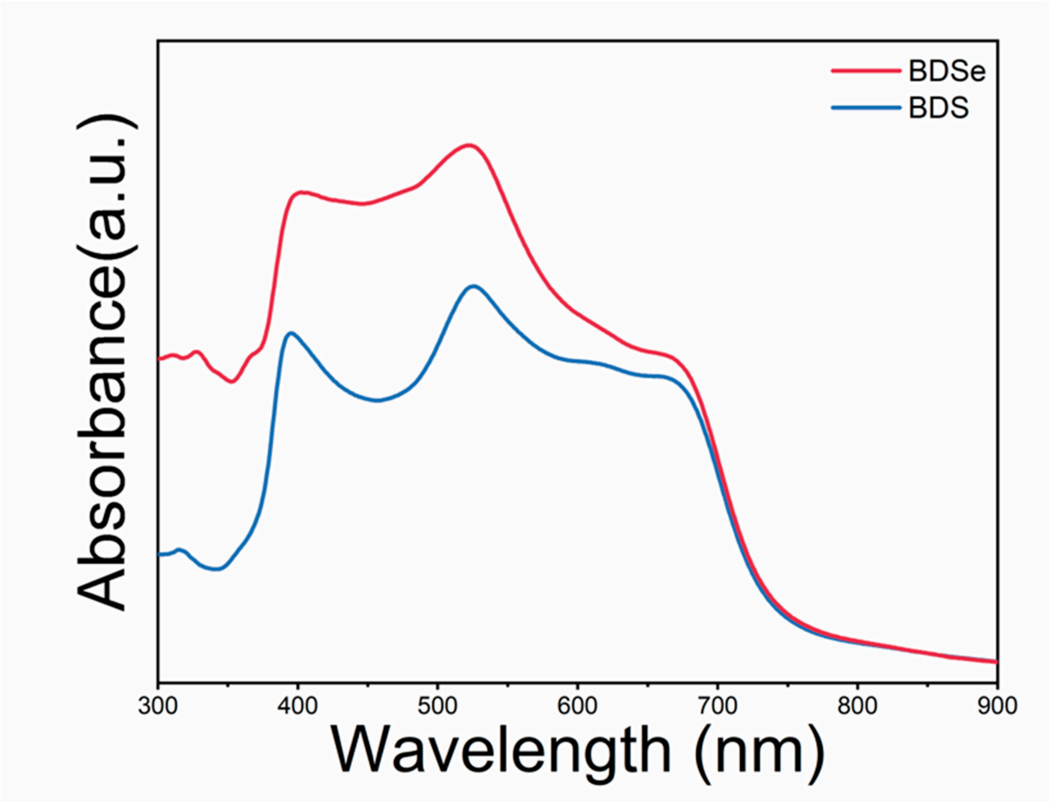

Figure S4. UV Visible Absorption Spectroscopy of BDS and BDSe Films.

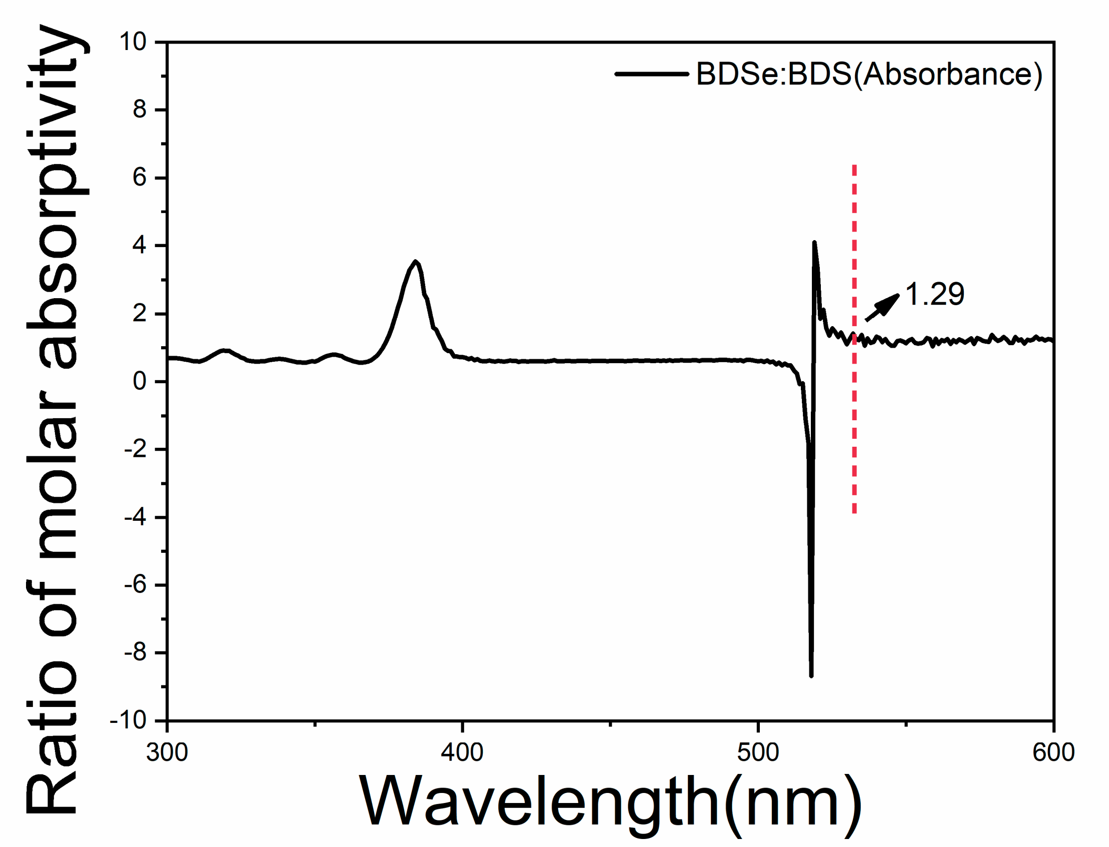

Figure S5. The ratio of molar absorptivity of BDSe and BDS.

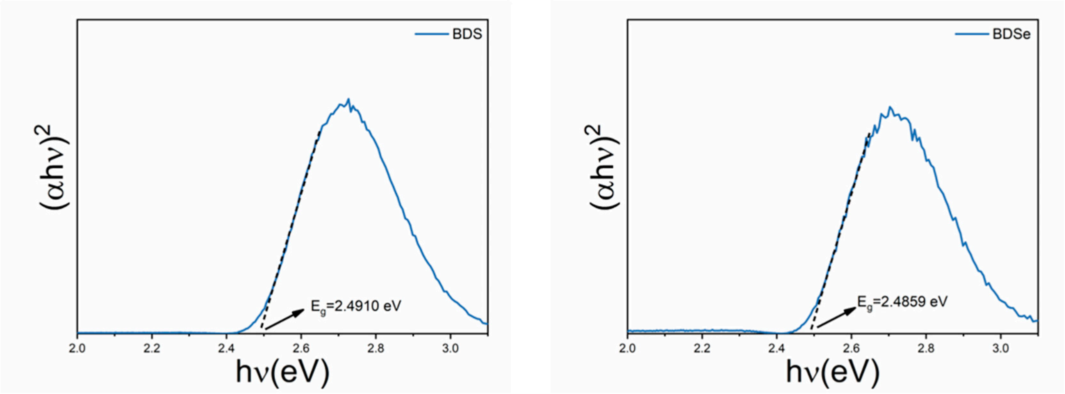

Figure S6. Tauc plots of BDS and BDSe.

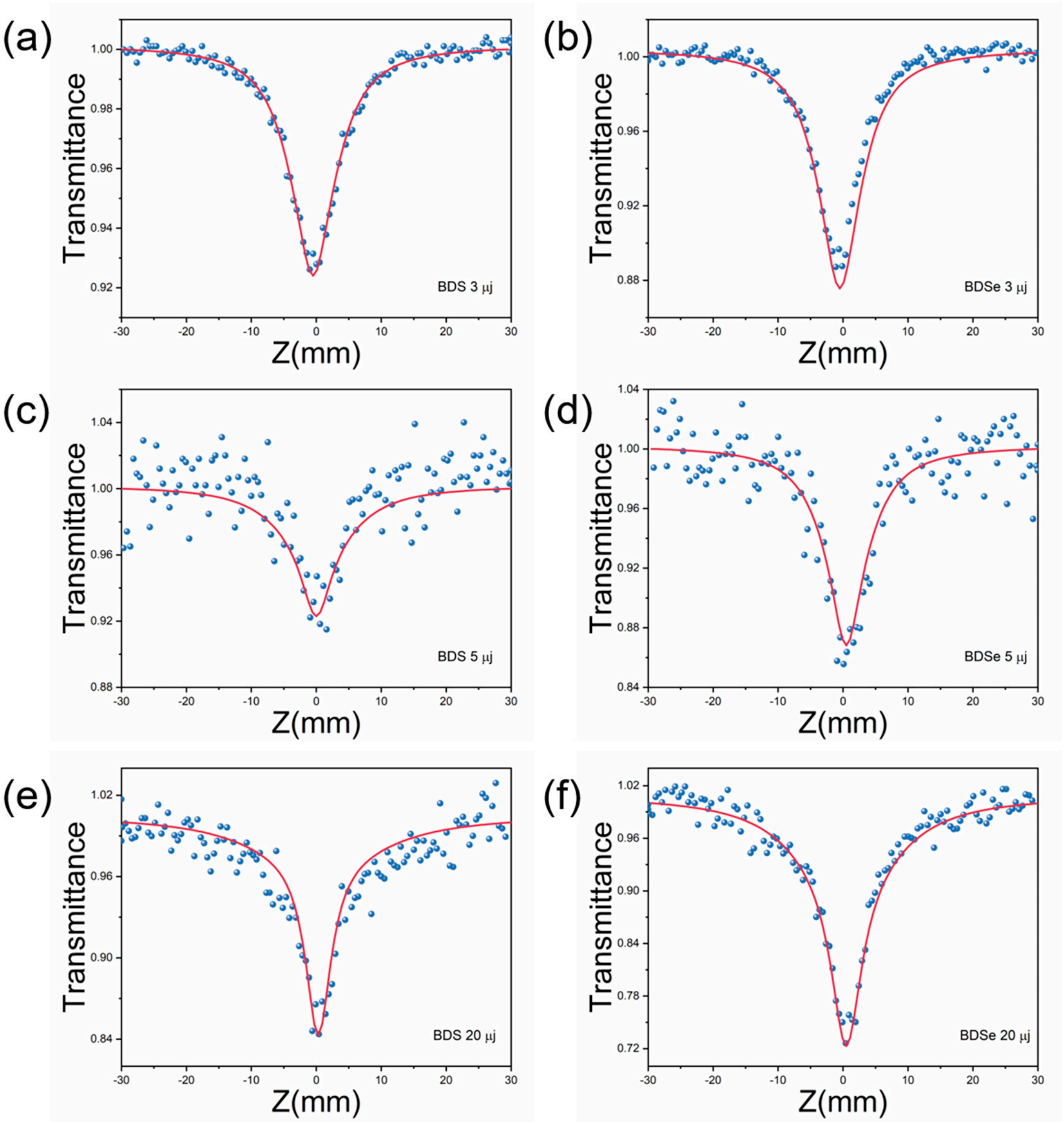

Figure S7. Z-scan fitting results of BDS and BDSe under laser excitation with ns pulse width.

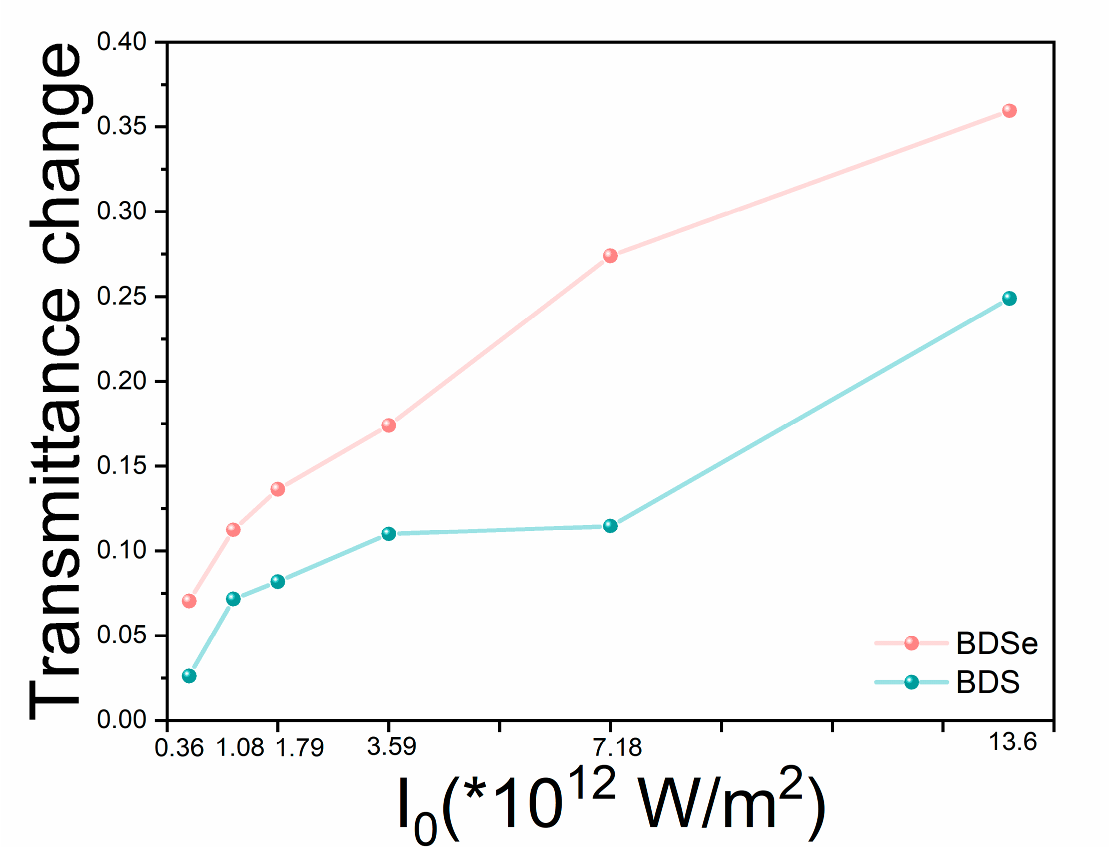

Figure S8. The relationship between peak transmittance change and light intensity (I_0) in Z-scan test results of BDS and BDSe.

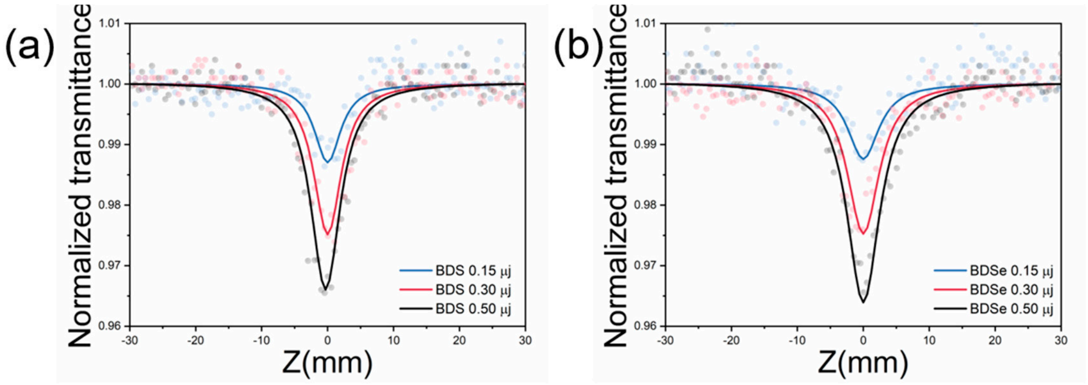

Figure S9. Z-scan test results of BDS and BDSe under excitation of ns pulse width laser with different energies.

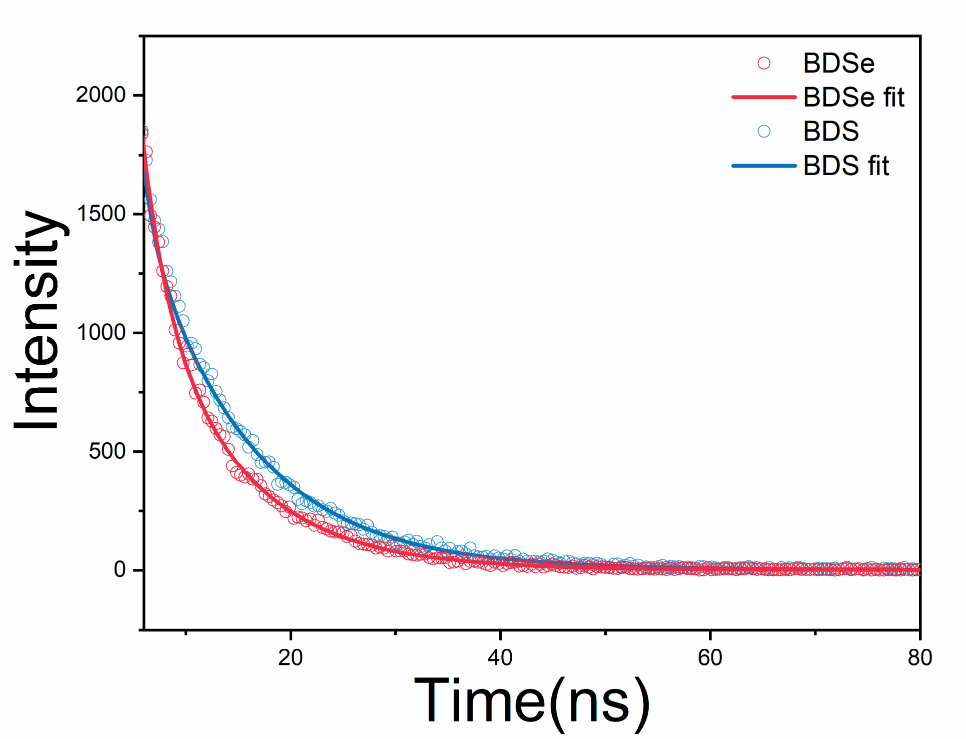

Figure S10. The fluorescence decay curves of BDS and BDSe.

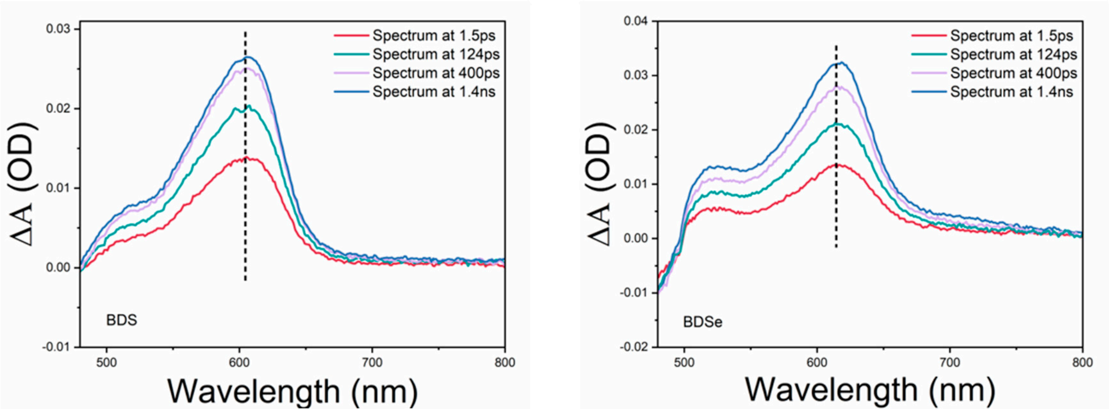

Figure S11. Transient absorption spectra of BDS and BDSe.

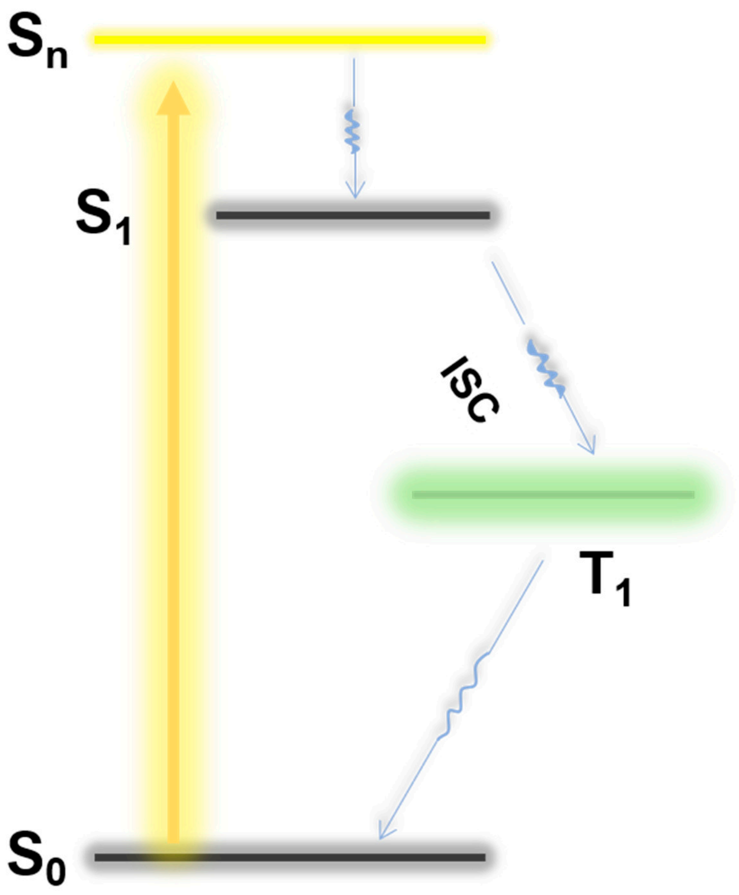

Figure S12. Schematic of typical photophysical processes.

Table S1. Electronic transition types of BDS/BDSe molecules and their corresponding excitation energy calculations.

molecule	Electronic transition	Energy (eV/nm)
BDS	$S_0 \rightarrow S_1$	2.5962/477.56
	$S_0 \rightarrow S_2$	3.4799/356.29
	$S_0 \rightarrow S_3$	3.5363/350.60
	$S_0 \rightarrow S_4$	3.6196/342.54
	$S_0 \rightarrow S_5$	3.7310/332.21
	$S_0 \rightarrow S_6$	4.0928/302.93
	$S_0 \rightarrow S_7$	4.2453/292.05
BDSe	$S_0 \rightarrow S_1$	2.5884/478.99
	$S_0 \rightarrow S_2$	3.4325/361.21
	$S_0 \rightarrow S_3$	3.4771/356.57
	$S_0 \rightarrow S_4$	3.5397/350.27
	$S_0 \rightarrow S_5$	3.7104/334.15
	$S_0 \rightarrow S_6$	3.9982/310.10
	$S_0 \rightarrow S_7$	4.1254/300.54

Table S2. The Cartesian coordinates of BDS.

C	0.67669	0.267197	-0.20431
C	-0.75883	0.221801	-0.24174
C	-1.49436	1.417577	-0.32627
C	-0.83487	2.680115	-0.21164
C	0.589509	2.711166	0.030589
C	1.325901	1.48731	0.006889
N	1.073121	3.928937	0.185356
N	-1.39341	3.875361	-0.22961
S	-0.1935	4.969301	0.038457
C	-2.85944	1.049558	-0.38276
C	-2.91876	-0.32845	-0.28325

N	-1.642	-0.85351	-0.18739
C	-5.17297	0.218079	-0.33128
C	-4.25602	-0.81424	-0.2461
S	-4.42654	1.802522	-0.44916
C	-6.53989	-0.20257	-0.32229
C	-6.61328	-1.55811	-0.22445
C	-7.70925	0.727974	-0.40475
H	-8.65317	0.178909	-0.37626
H	-7.70548	1.440116	0.427966
H	-7.68361	1.31117	-1.33201
C	-1.41703	-2.10225	0.548657
C	-1.06573	-1.89404	2.026521
C	-2.06807	-0.99006	2.739683
C	-0.97449	-3.25596	2.712192
H	-1.7989	-0.88761	3.795747
H	-2.09684	0.014159	2.308705
H	-3.0807	-1.40705	2.69343
H	-1.94714	-3.76221	2.70623
H	-0.2489	-3.91406	2.224263
H	-0.66735	-3.1415	3.756126
H	-0.07809	-1.41861	2.066417
C	2.711377	1.198415	0.042231
C	2.874939	-0.15628	-0.19075
N	1.620959	-0.75309	-0.36106
C	5.07257	0.560694	0.09229
C	4.256111	-0.53086	-0.16156
C	6.468071	0.266653	0.183174
C	6.676772	-1.06453	0.000229
C	1.396188	-1.78169	-1.39485
C	2.144309	-3.10005	-1.2027
C	1.667629	-4.08561	-2.26903
C	2.011269	-3.69129	0.197268
H	2.688867	-4.54261	0.315286
H	2.250385	-2.96214	0.976495

H	0.995408	-4.05782	0.375591
H	1.78469	-3.67618	-3.27798
H	2.239755	-5.01687	-2.21859
H	0.60945	-4.33605	-2.12843
H	3.203199	-2.91096	-1.39749
S	4.194036	2.059982	0.300063
C	7.527997	1.289755	0.446775
H	7.529975	2.06468	-0.32791
H	7.361926	1.790303	1.407417
H	8.520028	0.832986	0.471111
H	7.626051	-1.58388	0.008287
H	-7.51127	-2.16059	-0.18883
S	5.219204	-1.97286	-0.27873
S	-5.05964	-2.34621	-0.15022
H	0.323163	-1.96249	-1.43802
H	1.675491	-1.353	-2.36503
H	-2.34115	-2.67976	0.466752
H	-0.64156	-2.68843	0.060732

Table S3. The Cartesian coordinates of BDSe.

C	0.688197	0.530597	-0.24278
C	-0.74652	0.493367	-0.30084
C	-1.47391	1.696133	-0.38463
C	-0.80659	2.952905	-0.25574
C	0.615029	2.973183	0.004261
C	1.343024	1.744438	-0.01511
N	1.104502	4.186622	0.174043
N	-1.35705	4.152056	-0.2721
S	-0.15332	5.236238	0.018271
C	-2.84108	1.339487	-0.44349
C	-2.91022	-0.03788	-0.34586
N	-1.63862	-0.57283	-0.26036
C	-5.15805	0.513415	-0.31506
C	-4.24248	-0.51869	-0.25914
S	-4.4063	2.095562	-0.46997
C	-6.54076	0.152184	-0.2006
C	-6.70435	-1.18496	-0.03269
C	-7.65312	1.154394	-0.249
H	-8.62599	0.670879	-0.13473
H	-7.54781	1.897544	0.549574
H	-7.65314	1.697432	-1.20074
C	-1.43128	-1.87186	0.381995
C	-1.26263	-1.80868	1.906067
C	-2.34803	-0.98254	2.593576
C	-1.24307	-3.23661	2.449822
H	-2.22547	-1.0305	3.680248
H	-2.30561	0.070855	2.303281
H	-3.34781	-1.35818	2.349855
H	-2.21462	-3.72228	2.298637
H	-0.48017	-3.85313	1.963227
H	-1.03484	-3.23891	3.523882
H	-0.28935	-1.34367	2.107287
C	2.725484	1.441563	0.059771

C	2.879375	0.085582	-0.16289
N	1.626076	-0.50067	-0.37672
C	5.073157	0.75269	0.207152
C	4.243517	-0.32041	-0.07165
C	6.470679	0.480194	0.366013
C	6.746639	-0.84029	0.222454
C	1.433818	-1.48424	-1.4616
C	2.17917	-2.8121	-1.31296
C	1.628001	-3.79096	-2.34938
C	2.12077	-3.40326	0.093273
H	2.745646	-4.30011	0.152223
H	2.473101	-2.70131	0.853838
H	1.100279	-3.69773	0.356928
H	1.668523	-3.37173	-3.36043
H	2.205463	-4.72031	-2.35081
H	0.583856	-4.04639	-2.13327
H	3.227225	-2.63599	-1.57497
S	4.213228	2.271874	0.371593
C	7.481494	1.545141	0.662379
H	7.495666	2.306814	-0.1254
H	7.251672	2.05589	1.604469
H	8.485956	1.123401	0.743519
H	7.725298	-1.29563	0.296123
H	-7.64784	-1.70202	0.079317
H	0.363184	-1.66554	-1.54086
H	1.733989	-1.01224	-2.40515
H	-2.29829	-2.48609	0.122567
H	-0.56762	-2.36556	-0.0553
Se	-5.09566	-2.14538	-0.01152
Se	5.242099	-1.88895	-0.13365
